# Targeting Prolyl Endopeptidase with Valproic Acid as a Potential Modulator of Neutrophilic Inflammation

**DOI:** 10.1371/journal.pone.0097594

**Published:** 2014-05-16

**Authors:** Mojtaba Abdul Roda, Mariam Sadik, Amit Gaggar, Matthew T. Hardison, Michael J. Jablonsky, Saskia Braber, James Edwin Blalock, Frank A. Redegeld, Gert Folkerts, Patricia L. Jackson

**Affiliations:** 1 Division of Pharmacology, Utrecht Institute for Pharmaceutical Sciences, Faculty of Sciences, Utrecht University, Utrecht, The Netherlands; 2 Department of Medicine, Division of Pulmonary, Allergy and Critical Care Medicine and UAB Lung Health Center, University of Alabama at Birmingham, Birmingham, Alabama, United States of America; 3 Department of Chemistry, University of Alabama at Birmingham, Birmingham, Alabama, United States of America; Institute of Lung Biology and Disease (iLBD), Helmholtz Zentrum München, Germany

## Abstract

A novel neutrophil chemoattractant derived from collagen, proline-glycine-proline (PGP), has been recently characterized in chronic obstructive pulmonary disease (COPD). This peptide is derived via the proteolytic activity of matrix metalloproteases (MMP's)-8/9 and PE, enzymes produced by neutrophils and present in COPD serum and sputum. Valproic acid (VPA) is an inhibitor of PE and could possibly have an effect on the severity of chronic inflammation. Here the interaction site of VPA to PE and the resulting effect on the secondary structure of PE is investigated. Also, the potential inhibition of PGP-generation by VPA was examined *in vitro* and *in vivo* to improve our understanding of the biological role of VPA. UV- visible, fluorescence spectroscopy, CD and NMR were used to determine kinetic information and structural interactions between VPA and PE. *In vitro*, PGP generation was significantly inhibited by VPA. *In vivo*, VPA significantly reduced cigarette-smoke induced neutrophil influx. Investigating the molecular interaction between VPA and PE showed that VPA modified the secondary structure of PE, making substrate binding at the catalytic side of PE impossible. Revealing the molecular interaction VPA to PE may lead to a better understanding of the involvement of PE and PGP in inflammatory conditions. In addition, the model of VPA interaction with PE suggests that PE inhibitors have a great potential to serve as therapeutics in inflammatory disorders.

## Introduction

COPD is defined as a disease state characterized by airflow limitation that is not fully reversible. The chronic airflow limitation characteristic of COPD is caused by a mixture of small airway disease (obstructive bronchiolitis) and parenchymal destruction (emphysema), the relative contributions of which vary from person to person. The prevalence, morbidity, mortality and treatment costs of COPD are high and increasing. COPD is the third leading cause of death in the United States and the fourth worldwide [1], [2].

In COPD, multiple classes of proteases are released from neutrophils in the airway compartment, including endopeptidases, serine proteases, and matrix metalloproteinases (MMPs) [3]. Recently, Weathington et al have characterized a novel neutrophil chemoattractant, proline-glycine-proline (PGP) which is derived from collagen [3], [4]. An acetylated form of this peptide (N-α-PGP) is also detected and demonstrates increased chemotactic properties compared to non-acetylated PGP. PGP acts as a neutrophil chemoattractant *in vitro* and induces neutrophilic inflammation when instilled into the airways of mice in-vivo. PGP is known to act on CXC receptors 1 and 2 (CXCR1, CXCR2) on neutrophils due to a structural homology with ELR+ chemokines, such as interleukin-8 (IL-8). Chronic N-α-PGP administration into murine airways for 12 weeks at biweekly intervals leads to the development of neutrophilic airway inflammation, alveolar enlargement, and right ventricular hypertrophy, all of which are features of COPD. The degree of alveolar enlargement is similar to that seen with mice exposed to cigarette smoke 6 times per week for 24 weeks [3], [4]. Gaggar et al. have gone on to demonstrate a prominent role for this peptide in additional inflammatory neutrophilic lung conditions, such as cystic fibrosis (CF) and chronic allograft rejection after lung transplantation [3], [5].

Generation of PGP occurs via initial cleavage of collagen by matrix metalloproteases (MMP-8, MMP-9) and subsequently by prolyl endopeptidase (PE) [3]. This occurs when there is some initial insult to the epithelial layer, which leads to an exposure of collagen. It has been shown that all three enzymes, MMP-8, 9 and PE, are found in neutrophils and are present in COPD serum and sputum [6], [7]. PE is a protease that belongs to the serine protease family. This enzyme cleaves the carboxyl side of proline residues in oligopeptides [8].

Recently, PE has been described as part of the signaling pathways involved in phosphoinositides leading to neuronal cone growth in the brain [9]. This work was done in an attempt to determine the pathophysiological mechanism of the mood stabilizer drug valproic acid (VPA). VPA is used clinically as a mood stabilizer in mania, bipolar disorder, epilepsy, attention-deficit hyperactivity disorder (ADHD), chorea, and for migraine headaches. The recommended therapeutic plasma level is 312–693 µM [10]. In healthy volunteers, VPA has been shown to be highly protein bound (85–95%) after a single intravenous bolus dose [11].

Cheng et al. showed that VPA can directly inhibit recombinant human PE (rhPE) as well [12]. It is surprising that VPA is a specific inhibitor of PE, as it does not resemble the normal peptide substrates of PE to act as a transition state analogue, nor does it fit a classical serine protease inhibitor family [13].

Many compounds are known to have an inhibitory effect on PE, such as ZPP (N-carbobenzoxy-proline-prolinal), S-17092 (2S,3aS,7aS)-1((R.R)-2-phenylcyclopropyl)carbonyl)-2-((thiazolidin-3-yl)car-bonyl)octahydro-1H-indole) and JTP-4819 ((S)-2-(((S)-2-(hydroxyacetyl)-1-pyrrolidinyl)carbonyl)-N- phenylmethyl)-1-pyrrolidinecarboxamide) [14]-[16]. Yet, none of these compounds are registered as active drug compounds. VPA is the only drug compound we are aware of, that inhibits PE and is also approved to be given to patients [12], [17].

Thus, there is an increased need for knowledge regarding the structure of VPA bound to PE and the exact site or sites of binding on PE. To this end, we have undertaken studies to elicit data to further this knowledge through the use of CD and NMR. We have also shown a direct inhibitory effect of VPA on a system generating the matrikine PGP likely through inhibition of PE.

## Methods

### PE activity assays

The PE activity assay was performed with specific PE substrates (both purchased from Bachem, Switzerland):

N-succinyl-glycine-proline-*para-nitroaniline* (Suc-Gly-Pro-pNA) and N-succinyl-glycine-proline-7-amido-4-methyl-coumarin (Suc-Gly-Pro-AMC). Lithium (Sigma-Aldrich, USA) and VPA (Sigma-Aldrich, USA) were used as a competitor.

Recombinant human PE was expressed in E. coli using the plasmid pTrcHis PE, kindly provided by dr. A.W. Mudge, in Promega BLR1(DE3)pLys 3 E. coli competent cells as previously reported [12].

Activity assays were carried out in 100 mM phosphate buffer (pH 7.5). 1 mM DL-Dithiothreitol (DTT) (Sigma-Aldrich, USA) and 10 µM bovine serum albumin (BSA) (Sigma-Aldrich, USA) were added and left over night at 4°C. The reactions were performed in a final volume of 100 µl with a final rhPE concentration of 10 nM.

PE-specific fluorogenic substrate Suc-Gly-Pro-AMC (0.2 mM) was used to do a lithium/VPA-dose response (0–10 mM), measured with a spectrofluorometer using excitation and emission wavelengths of 380 nm and 460 nm respectively, at 37°C over 60 min.

PE-specific colorgenic substrate Suc-Gly-Pro-pNA (0–10 mM) was used to do a substrate dose response with three VPA concentrations: 0.8, 1.6 and 3.5 mM. PE-activity was measured with an Ultra violet- visible (UV/Vis) spectrometer at a wavelength of 405 at 37°C during 60 min. Suc-Gly-Pro-pNA and Suc-Gly-Pro-AMC are both water soluble and were dissolved in phosphate buffer. In all cases, VPA was pre-incubated with the enzyme at 37°C for 90 min.

### Collagen digestion with PMN lysate

Polymorphonuclear neutrophils (PMNs) were isolated from a buffy coat of normal human blood donors (Research Blood Components). The buffy coat (30 ml) was diluted with PBS to 250 ml. PMNs were separated as described before [5] **.** Briefly, the diluted buffy coat was separated by a Histopaque gradient (Sigma-Aldrich, St. Louis, MO). This step was repeated once more to remove the final inclusion of erythrocytes. PMNs were counted on a Hemacytometer slide after staining with Trypan blue (Sigma-Aldrich, USA).

Neutrophil lysate was obtained by two freeze–thaw cycles with 10 µg/ml aprotinin (Sigma-Aldrich) and 10 µM BSA, followed by centrifugation at 2000 x g at 4°C for 10 min. Lysates were left on ice for one hour, and incubated hereafter with bestatin (Cayman Chemical, Ann Arbor,MI) and one of the following: PBS, 5, 10, 50 or 100 mM VPA at 37°C for 30min. The lysate/VPA mixtures were then mixed with predialyzed Collagen Type I and II (Sigma-Aldrich). Final concentrations for both types were 0.11 mg/ml. From each sample 75 µl was taken to measure PE activity. The samples were then left on a shaker for 20 hours at 37°C. Every 5 hours, bestatin was re-added, to reach a final concentration of 0.9 mg/ml. Negative controls contained 100 mM VPA added to collagen alone and were treated the same as any the other sample (n = 10 per group).

### Animals

Female A/J mice, 7–9 weeks (Jackson Labs) old were housed under controlled conditions in standard laboratory cages. They were provided free access to water and food. All *in vivo* experimental protocols were approved by the UAB Institutional Animal Care and Use Committee

### Cigarette smoke exposure

Mice were exposed in whole-body chambers (5 L) to air (sham) or to 40 times diluted mainstream cigarette smoke during 5 consecutive days using a SIREQ (Tempe, AZ, USA) smoking device. Reference cigarettes 2R4F (University of Kentucky, Lexington, Kentucky) were puffed at 3 L/min. Just before the exposure, filters were cut off from the cigarettes. Each cigarette was smoked in 10 minutes rounds (1 puff/min). Per round 2 cigarettes were smoked. Mice were exposed twice daily to cigarette smoke using 4 cigarettes per exposure on days 1–2, and 6 cigarettes on days 3–5. The mice administered either vehicle (70 µl PBS) or VPA (100 µg in 70 µl sterile PBS) by oropharyngeal aspiration under light isoflurane anesthesia twice daily before each smoke exposure. The mice were sacrificed 16 hours after the last air or smoke exposure as described before [18].

### Bronchoalveolar lavage

Immediately after i.p. injection with an overdose of ketamine/xylazine mix, the lungs of the mice were lavaged 4 times through a tracheal cannula with 1 ml PBS, pre-warmed at 37°C. After centrifuging the BAL fluid at 4°C (400 g, 5 min), the cell pellets of the 4 lavages were used for cell counts. The 4 cell pellets, kept on ice, were pooled per animal and resuspended in 150 µl cold saline. The supernatant of the first 1 ml lavage was used to measure PGP after mixing with 1 mM bestatin. After staining with Türk solution, total cell counts per lung were made under light microscopy using a Burker-Turk chamber. Differential cell counts were performed on cytospin preparations stained by DiffQuick (Dade A.G., Düdingen, Switzerland). Cells were identified as macrophages, neutrophils and lymphocytes according to standard morphology. At least 200 cells were counted and the absolute number of each cell type was calculated [18].

### CD spectroscopy

CD spectra were obtained of VPA (6, 12 and 24 mM) or ZPP (250, 500 and 1000 nM) titrated into 2.5 µM rhPE in PBS. Also, 24 mM VPA was added to PE:ZPP (2.5 µM : 1 µM) and 1 µM ZPP (Enzo) was added to PE:VPA (2.5 µM : 24 mM). PE was solubilized in PBS, VPA in H_2_O and ZPP in 0.006% (v/v) DMSO (in H_2_O). Samples were injected in a 0.5 mm cell and measured with a Jasco J-815 CD-Spectrometer at wavelengths ranging from 260–194 nm (data pitch 0.5 nm). All samples were analyzed on the same day with the same sample stocks, in order to obtain reliable results.

### NMR spectroscopy

^1^H 1-D NMR was carried out on a Bruker Avance 700 MHz NMR spectrometer using a 5 mm cryoprobe operated at 20°C. Samples of enzyme (PE) were run in a Shigemi microcell. The concentration of PE was approximately 10 µM in 250 µl PBS (90% H_2_O; 10% D_2_O). The residual water signal was suppressed using presaturation. ^1^H NMR spectra were obtained for 10 mM VPA (in PBS (95% D_2_O)) or 10 µM ZPP (<0.001% (v/v) DMSO (PBS (95% D_2_O)) in the presence or absence of the enzyme. Also, 10 mM VPA was added to PE:ZPP (10 µM : 10 µM) and 10 µM ZPP was added to PE:VPA (10 µM : 10 mM).

### PGP measurement

Neutrophil lysate with collagen samples were 10 kDa filtered and analyzed by ESI-LC-MS/MS for PGP, as described before [5]. BAL fluid was analyzed utilizing the same method without a filtration step.

During all experiments the pH of all solutions was measured before and after adding the PE inhibitors to PE in PBS. The pH of all solutions did not drop under 7.0 after adding the inhibitors.

## Results

### PE activity

**Figure 1:**
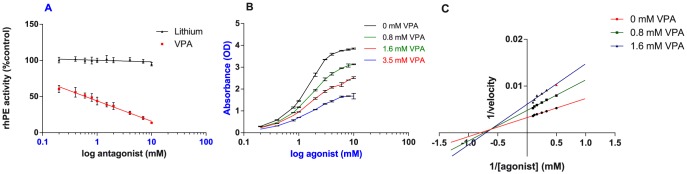
Effect of VPA on the activity of rhPE. (**A**) Activity of 10 nM purified rhPE was measured in presence of ten doses of VPA or lithium ranging from 0.2–10 mM. Relative activity of rhPE in the presence of VPA or lithium is shown as a percentage of activity in the absence of VPA and lithium. VPA showed a *K_i_* of approximately 1 mM. lithium showed no effect on PE activity. (**B**) Inhibition curves of three VPA concentrations (0.8, 1.6 and 3.5 mM) were obtained by incubating VPA with 10 nM rhPE during 90 min at 37 °0. (**C**) A Lineweaver-Burk plot was made based on the rhPE activity assays with 0.8 and 1.6 mM VPA as inhibitor. Enzyme activity was measured with increasing substrate (Suc-Gly-Pro-pNA) concentrations, ranging from 0.2–10 mM. The velocity was calculated as mM * min^−1^ * ml^−1^. Data are shown as the mean ± S.E.M. (n = 3 per group).

VPA inhibited PE in a dose dependent way, with a *K_i_* of approximately 1–2 mM, as seen in figure 1A and B. Lithium, a drug used in bipolar disease as well, showed no effect on the activity of PE at the same dose range as VPA (0–10 mM), similar to the findings of Cheng et al [12]. Increasing VPA concentrations was shown to decrease the *V_max_* of the enzyme, in a non-competitive way. 1.6 mM VPA lowered the *V_max_* of the enzyme to 0.21 mOD * min^−1^ * µl^−1^ compared to a *V_max_* of 0.40 mOD * min^−1^ * µl^−1^ in the control group. However, fitting the data in a Lineweaver-Burk plot, a modified form of the Lineweaver-Burk form was obtained repeatedly, as shown in figure 1C. This confirms that VPA inhibits PE in a mixed non-competitive way, as published before [12].

### PGP generation

**Figure 2:**
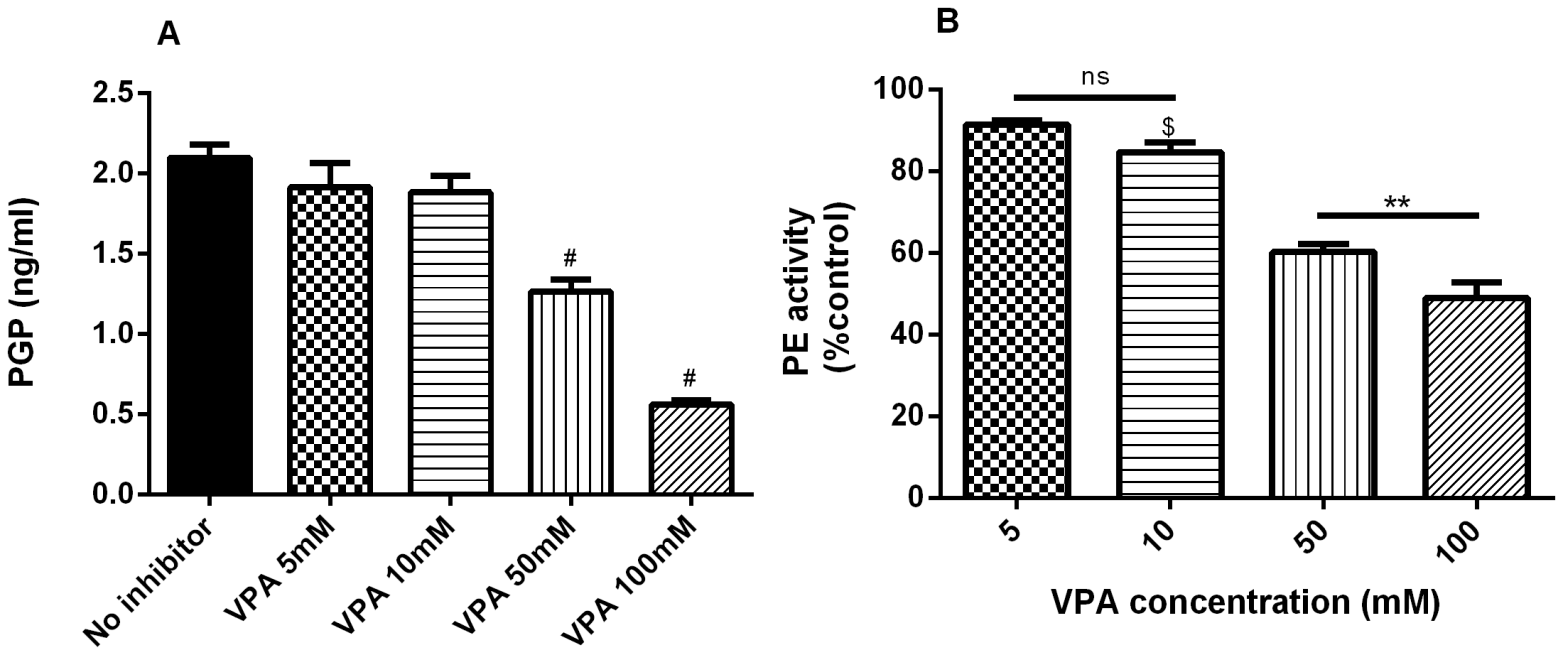
Inhibition of PGP generation by VPA. (**A**) Dialyzed Collagen Type I and II were incubated with the lysate of 4.6 * 10^6^ PMN at 37°C for 20 hours to generate PGP. (**B**) PE activity was measured 30 minutes after incubation of lysate/collagen/VPA mixture and compared to control (no VPA). Data are shown as the mean ± S.E.M. (n = 5–10 per group). Representative of 4 experiments. # p<0.0001 compared to control, $ p<0.01 compared to control, ** p<0.01, ns p>0.05.

Using the lysates of PMNs, collagen was digested to generate PGP in the presence or absence of VPA, as seen in figure 2A. In the absence of a PE inhibitor, PGP levels of up to 2.1 ng/ml were obtained with negative controls providing a baseline measurement. A total VPA concentration of 50 and 100 mM inhibited the PGP generation from collagen Type I and II significantly (p<0.0001). 5 and 10 mM total VPA showed inhibition though not reaching significance, p = 0.14 and p = 0.052 respectively. The PE activity showed a significant decline with 10, 50 and 100 mM VPA (figure 2B).

### Cigarette smoke exposure

**Figure 3:**
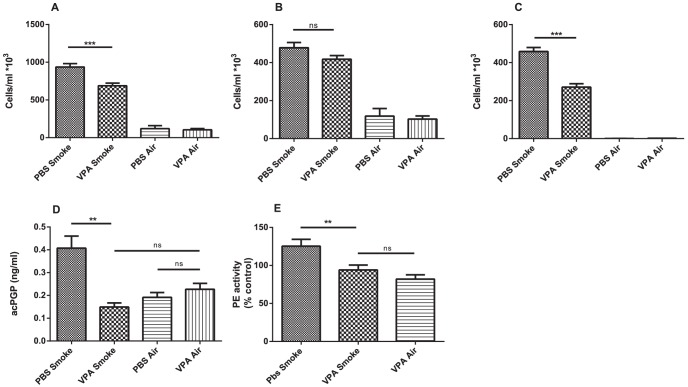
VPA decreases cigarette smoke-induced neutrophil influx in BAL fluid of mice. (**A**) Total cell numbers, (**B**) macrophages and (**C**) neutrophils in the BAL fluid of mice exposed to air or whole body cigarette smoke twice daily during 5 days. The mice received vehicle (PBS) or VPA (100 µg/70 µl PBS) by oropharyngeal aspiration 15 minutes prior to air/smoke exposure. (**D**) acPGP was measure in the BAL fluid. (**E**) PE activity was measured in the BAL fluid and compared to control (PBS treated/air exposed mice). N = 5–10 animals per group. Values are expressed as mean+/−S.E.M. **P≤0.01, ***P≤0.001, ns p>0.05.

A significant increase in BAL fluid neutrophils and macrophages was observed after 5 days cigarette smoke exposure compared to the air-exposed mice (figure 3A-C). Unlike the macrophages, cigarette smoke-induced neutrophil influx in the BAL fluid was significantly decreased after VPA administration. Accordingly, the acPGP levels and the PE activity were the highest in the BAL fluid of the PBS treated smoked mice. The acPGP levels and the PE activity of the other groups was not significantly different from each other (Figure 3D-E).

### CD spectroscopy

**Figure 4:**
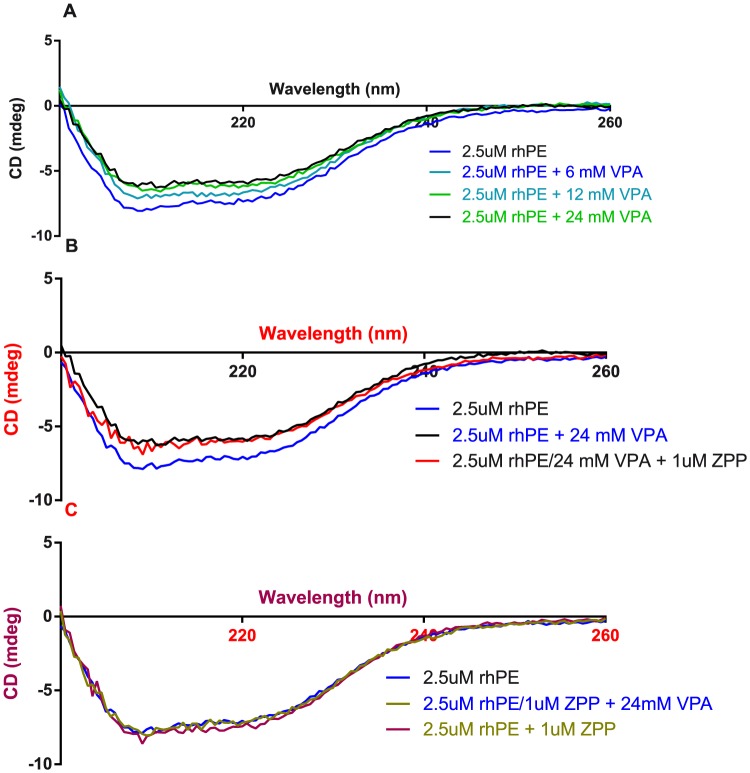
Effect of VPA on the secondary structure of rhPE. (**A**) Three VPA concentrations (6, 12 and 24 mM) were titrated into 2.5 µM rhPE. VPA caused a significant change in the secondary structure of the enzyme. (**B**) A high concentration of ZPP (1 µM) was added to the 2.5 µM rhPE/24 mM VPA mixture. ZPP did not cause any change in the secondary structure of the enzyme. (**C**) Adding 1 µM ZPP to 2.5 µM rhPE does not cause any secondary protein structure change. Adding 24 mM VPA on top of that mixture doesn't cause any structure change.

Titrating VPA into PE resulted in a loss in signal compared with the same dilution with PBS added to 2.5 µM PE (figure 4A). Adding up to 1 µM ZPP (1000 times *K_i_*) on top of the PE:VPA mixture, did not give any additional change in the secondary structure of the enzyme (figure 4B). When adding 1 µM ZPP to PE, no change was observed to the PE secondary structure compared to the same dilution with PBS. Adding up to 24 mM VPA on top of the PE:ZPP mixture did not show any change in signal. These results suggest that VPA changes the secondary structure of rhPE by binding at or near the binding pocket of rhPE.

### NMR spectroscopy

**Figure 5:**
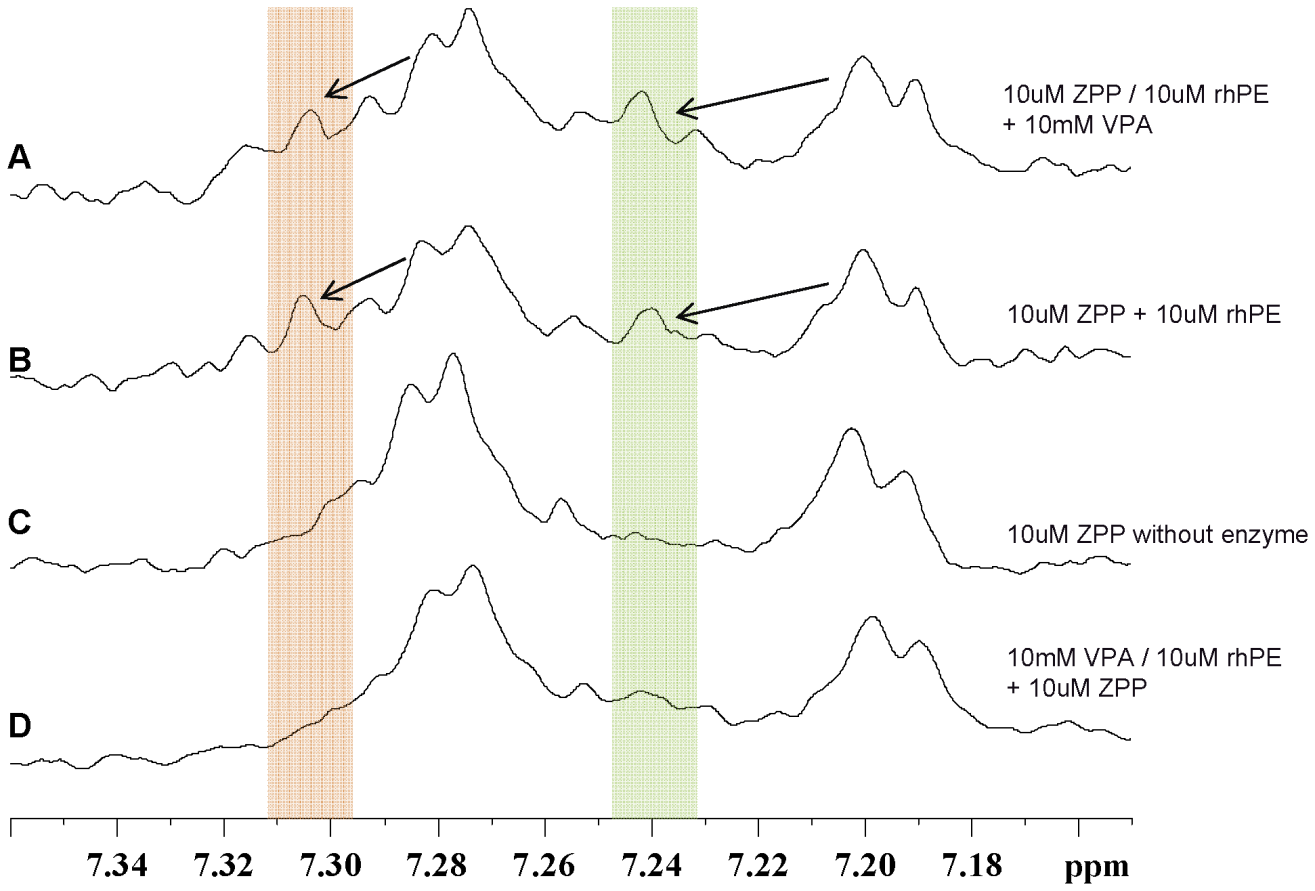
NMR spectra of the carbobenzoxy-group of ZPP. (**A**) 10 µM ZPP was added to 10 µM rhPE, 10 mM VPA was added to that mixture. A shift of the free ZPP peaks at 7.28 and 7.20 ppm to the left is seen (indicated in the red and green bars respectively). (**B**) 10 µM ZPP was added to 10 µM rhPE. A shift of the free ZPP peaks at 7.28 and 7.20 ppm to the left is seen. (**C**) 10 µM ZPP was measured without enzyme. The peaks at 7.28 and 7.20 ppm are the free ZPP fractions. (**D**) 10 mM VPA was added to 10 µM rhPE, 10 µM ZPP was added to that mixture. Note that the free ZPP peaks at 7.28 and 7.20 ppm do not show a shift to the left; there are no peaks in the highlighted areas.

NMR showed that when ZPP bound to PE (figure 5B), the carbobenzoxy peaks, 7.20 and 7.28 ppm seen with free-ZPP (figure 5C), decreased in intensity with a concurrent appearance of new peaks at 7.24 and 7.35 ppm respectively. Adding 10 mM VPA on top of PE:ZPP (10 µM:10 µM) did not change the carbobenzoxy peaks (figure 5A). However, there was no appearance of these new peaks at 7.24 and 7.35 ppm when 10 mM VPA was added to the enzyme first with subsequent addition of ZPP (figure 5D).

## Discussion

In this report, we identified the manner of binding of VPA to PE and demonstrate an effect on the secondary structure of PE by VPA. Also, we demonstrate a novel aspect of VPA as a potential modulator of inflammation through its actions as an inhibitor of PE and PGP generation. The latter indicates that VPA has an anti-inflammatory effect and may serve as a lead compound to design PE inhibitors. One concern is the high concentrations VPA needed to inhibit PE activity in this study might lead to toxic side effects. Though the mechanism of VPA teratogenicity is currently unknown, this toxicity maybe altered through combination with compounds such as resveratrol, vitamin E and N-acetylcysteine (NAC) [19], [20]. The latter is a particularly appealing combination as NAC at high concentrations has also been shown effective in treatment of COPD [21].

VPA is known to be a highly protein-bound drug (85–95%) in serum [11]. Since cell lysate contains a high quantity of proteins, other than PE, it is therefore uncertain how much unbound VPA is available to inhibit the enzyme. Maes M et al. investigated PE activity before and after a sub-chronic VPA treatment of 14 days (range 12–23 days) in manic patients who did not take any drugs seven days prior to treatment. A mean dose of 1400 mg (±592) VPA/day reduced the PE-activity significantly (p = 0.02) [17]. This demonstrates that despite the high protein binding of VPA sufficient drug can be available to effectively inhibit enzyme activity.

Recently, VPA has shown a remarkable effect on MMPs by their action on histone deacetylase. 2 mM of VPA decreases MMP-2 and MMP-9 expression levels and thus its activity significantly. Tissue-specific inhibitor 1, the natural inhibitor of MMPs, showed enhanced expression levels (p<0.05) when G361, SKNMC or U87MG cells were treated with 1 mM VPA during 24 h [22]. At this time there is no evidence to suggest that VPA has a direct inhibitory action on MMPs thus the inhibitory effects of VPA in neutrophil lysates is likely due to its direct action on PE as was demonstrated in figure 2B.

We had our concerns that VPA being an acid, might influence PE by lowering the pH. That is why the pH of all solutions was measured before and after adding VPA. Little to no effect on the pH was observed caused by VPA: the pH never dropped under 7.0.

*In vivo* a clear inhibitory effect of VPA administration on cigarette smoke-induced neutrophil influx into the BAL fluid is shown in this study. Those results were backed up by decreased acPGP levels and reduced PE activity in the BAL fluid of VPA treated mice which were exposed to cigarette smoke. These results are in agreement with the importance of PE in the PGP generation. As shown before, PGP induces neutrophil chemotaxis, and decreasing PGP levels results in lower neutrophil infiltration and less alveolar enlargement [3], [4].

With CD spectrometry, VPA clearly demonstrated an effect on the secondary structure of rhPE. This effect results in a decrease in PE signal at 220 nm. The effect of VPA on PE secondary structure could not be explained by dilution of the enzyme, nor did we observe any indication of precipitation of PE that could account for this signal change. ZPP, a competitive slow-binding PE inhibitor, is known to act on the active site of the enzyme [23], [24]. Thus, VPA was compared to ZPP both singularly and in an additive relationship to elucidate whether the VPA effects were at the active site. Adding ZPP to the enzyme prior to adding VPA to the ZPP:PE mixture did not affect secondary structure of the enzyme as compared to VPA alone (Fig. 4). This clearly suggests that VPA is interacting at or near the binding pocket of PE and not through non-specific binding to the exterior of the molecule.

In order to further examine the exact mechanism underlying the effects of VPA on the secondary structure and activity of PE, we carried out NMR studies. PE, with a molecular weight of almost 80 kDa is too large for direct measurements by 1H or 13C NMR and would require labeling with 15N,13C isotopes. Thus we carried out studies to look at the 1H shifts of ZPP and VPA in the presence of PE. We compared the interaction of each inhibitor with PE in presence or absence of the other inhibitor. When ZPP is added to PE, the protons of the carbobenzoxy group on ZPP are detected at 7.28 and 7.20 ppm (figure 5B). Compared with the free ZPP peaks (figure 5C), the bound ZPP peaks show a decreased signal, with an increasing growth of new peaks at 7.34 and 7.25 ppm respectively (figure 5B). This was somewhat surprising but suggests that a slow exchange between free ZPP and the bound ZPP. When VPA was added to the PE prior to the addition of ZPP, the only peaks observed are for the unbound ZPP. These results suggest that ZPP was unable to reach the active site of the enzyme, due to the effect of VPA on the enzyme. VPA peaks (methyl-groups) were seen between 0.68–0.70 ppm (data not shown) and did not interfere with the carbobenzoxy peaks of the ZPP. There is a significant overlap of VPA peaks and the residual non exchanging peaks of PE in this region therefore we were unable to determine the effects of addition of ZPP on the VPA peaks.

Recent descriptions of the anti-cancer properties of VPA especially in cigarette smoke induced lung cancers are leading to the development new pro-drugs based upon VPA [25]. Our data suggest that a bound structure of VPA and its derivatives could benefit this new drug development. Given the worldwide burden of morbidity and mortality associated with inflammatory airway disorders, the use of VPA or its derivatives to target extracellular PE activity and subsequent Ac-PGP generation may have notable changes in disease phenotypes. More importantly, the successful targeting of this protease in the COPD airway may improve disease related clinical outcomes such as exacerbation rates, improvement in lung function decline over time, airway mucociliary transport, and quality of life indices.
